# Anthropometry of the medial femoral condyle in the Chinese population: the morphometric analysis to design unicomparmental knee component

**DOI:** 10.1186/s12891-021-03979-2

**Published:** 2021-01-20

**Authors:** Feifan Lu, Xiaowei Sun, Weiguo Wang, Qidong Zhang, Wanshou Guo

**Affiliations:** 1 China- Japan Friendship School of Clinical Medicine, Peking University, 100029 Beijing, China; 2 Graduate School of Peking Union Medical College, Chinese Academy of Medical Sciences, 100029 Beijing, China; 3 Department of Orthopedic Surgery, Beijing Key Lab Immune-Mediated Inflammatory Diseases, China-Japan Friendship Hospital, Peking Union Medical School, 100029 Beijing, China

**Keywords:** Anthropometry, Medial femoral condyle, UKA components, Chinese population

## Abstract

**Background:**

This study aimed to assess the radii of the distal and posterior articular surfaces of the medial femoral condyle in a Chinese population and provide detailed parameters of the knee joint for the future design of UKA components.

**Methods:**

This study included 500 consecutive Chinese patients who underwent knee MRI from Jan 2019 to Jan 2020. The two most appropriate circles were used to reveal the distal and posterior joint surfaces in the sagittal plane of the MRI images. The radius of the circle representing the distal articular surface in the sagittal plane was measured as R1, and the radius of the posterior articular surface was measured as R2. The distance between the centers of the two rotation circles was recorded as d. An independent t test was used to compare the differences between men and women. The Pearson correlation coefficient was calculated to analyze the correlation between R1 and R2. SPSS v19.0 software was used for statistical analysis.

**Results:**

The average values of R1, R2, R1/R2 and d were calculated. Scatter plots were constructed to show the trend of changes in the radius of the distal and posterior articular surfaces of the femoral condyle. R1, R2 and d differed significantly between men and women (*p* < 0.05). Correlation analysis showed that R1 was positively correlated with R2 (*r* = 0.61, *p* < 0.05).

**Conclusions:**

The data of the radii of the distal and posterior articular surfaces of the medial femoral condyle were provided. In the UKA design, the relationships between the radii of the distal and posterior articular surfaces should be taken into account.

## Background

Unicompartmental knee arthroplasty (UKA) is an appropriate therapeutic technique for medial compartment osteoarthritis (OA) of the knee joint, which has attracted increasing attention in recent years. UKA can effectively relieve pain and improve function by surface replacement in the lesion compartment. It has the advantages of minimal trauma and rapid recovery, and the long-term and medium-term curative effects are satisfactory [1–3]. However, most UKA components currently used in China are designed for the physique of Western individuals, and many studies have shown that components designed for Western patients are not matched for other populations [4–6]. Although current studies have shown that UKA has good clinical results in Asian population [7–9], it is still worth considering to design more suitable UKA components for Chinese people. Currently, the measurement of medial femoral condyle parameters is unsatisfactory, and insufficient knee joint parameter data are available to support the design of a UKA component suitable for use in Chinese populations. The current studies focus mainly on measurements of the knee joint related to total knee arthroplasty (TKA), and research on the size and shape parameters of the knee joint related to UKA is scarce.

In our previous study, we measured the anteroposterior and mediolateral dimensions of the medial tibial plateau in a Chinese population. It was concluded that the design of the UKA tibial component currently has defects [10]. Moreover, not only the tibial component but also an appropriate femoral component is highly important to maintaining the natural rotation and flexion of the knee joint in the process of movement [11, 12]; thus, it is important to design a UKA femoral component with optimal suitability for the knee joint after UKA. Previous studies have suggested that the surface of the medial femoral condyle is not a normal spherical surface [13–15]. The rotation radius of the posterior articular surface of the medial femoral condyle is usually smaller than that of the distal femur. However, currently, the most commonly used Oxford UKA femoral component is based on a spherical surface with a single rotation center [16]. This difference may adversely affect the kinematics of the knee joint after UKA.

Considering that a well-matched UKA femoral component is indeed important, we sought to obtain anthropometric measurements of the posterior and distal joint surfaces of the femoral condyle in the Chinese population. Therefore, the aim of our research was to assess the anatomical parameters of the medial femoral condyle in a Chinese population, analyze the correlations between the posterior articular surface and distal surface, and analyze the differences in the knee joint between men and women. To explain the variability in all normal populations, more samples should be examined to identify other possibly different anatomical aspects of the “general” model. There are many imaging methods to observe the knee joint, including X-ray, CT, Magnetic resonance imaging (MRI) and ultrasound [17, 18]. MRI was chosen for this study because it provides better definition of soft tissues such as articular cartilage, ligaments, tendons, muscles and capsules [19].

We expected that the measurement results would show that the shape and size of the Oxford UKA femoral component differ from those of the medial femoral condyle, information that will help to improve the design of the UKA femoral component in the future and provide detailed parameters of the knee joint in the Chinese population.

## Methods

This study included 500 consecutive Chinese patients who underwent knee MRI in our institute between Jan 2019 and Jan 2020: 250 men aged 19–55 years, with an average age of 37.5 (± 8.8) years, and 250 women aged 21–55 years, with an average age of 37.2 (± 8.3) years. Two groups were established according to sex. Inclusion criteria: Adults, normal development, no deformity of the knee joint; and no knee joint changes caused by knee joint disease and trauma. Exclusive criteria: valgus and valgus; more obvious knee injury; rheumatoid arthritis; and ankylosing spondylitis and other inflammatory arthritis patients [10].

The Institutional Review Board of China-Japan Friendship Hospital approved this study, and the ID number of the approval was 2013-SF-1.

Sample size was estimated to be 384 when the acceptable error is 0.3, Z_0.05_ value is 1.96 and overall standard deviation was estimated to be 3. Taking into account the easy availability of samples and the impact of sampling errors, 500 patients were included in the study.

The MRI equipment and parameter information are described in our previous publication [10]. Patients were placed in the supine position with the knee joint extended. The coronal plane, sagittal plane and transverse plane were scanned.

**Fig. 1:**
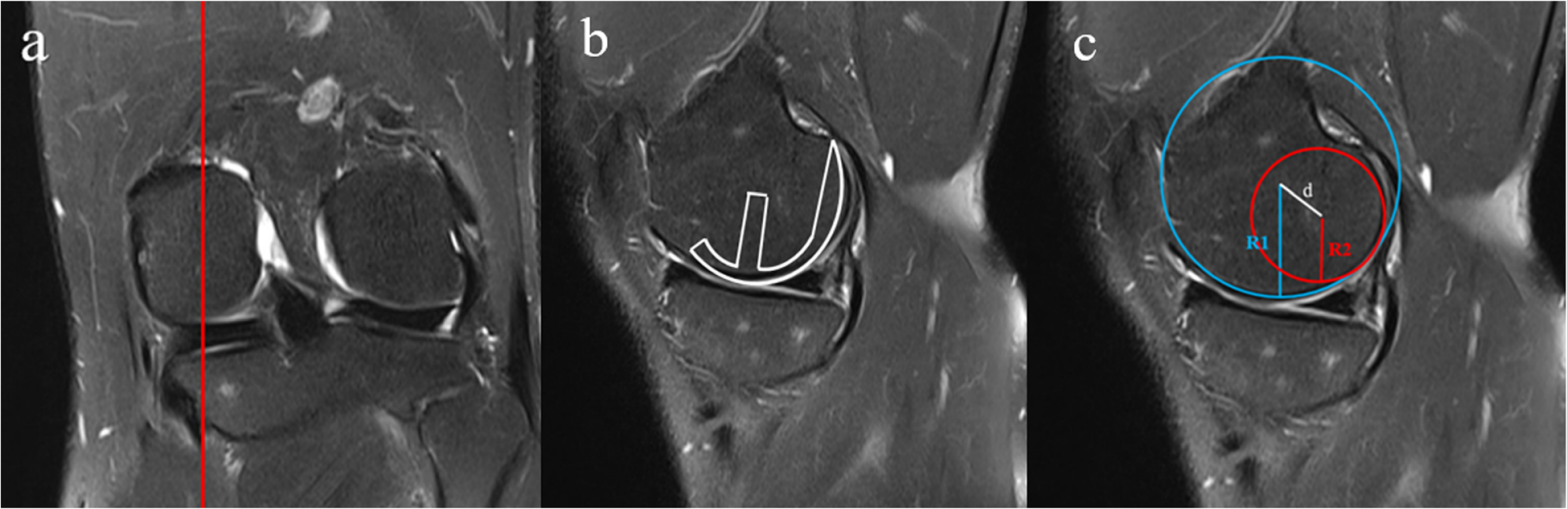
Medial femoral condyle measurement via MRI. **a** Measurement location in the middle of the femoral condyle in the coronal image. **b** Diagram of the location of the Oxford UKA component. **c** The two most appropriate circles were used to reveal the distal and posterior joint surfaces. The radius of the circle representing the distal articular surface was measured as R1, and the radius of the posterior articular surface was measured as R2. The distance between the centers of the two circles was recorded as d

The level of the MRI image was selected as follows: First, the coronal image was located in the middle of the femoral condyle (Fig. 1a), and the sagittal image in this plane, which could be considered the center position of the Oxford UKA component, was selected for analysis (Fig. 1b). In the sagittal plane of the selected surface (Fig. 1c), the two most appropriate circles were used to reveal the distal and posterior joint surfaces. Measurement of the sagittal profile with two circular arcs representing the medial condyles of the distal femur was described by Nuno [20]. After fitting, the radius of the circle representing the distal articular surface was measured as R1, and the radius of the posterior articular surface was measured as R2. The distance between the centers of the two circles was recorded as d. The measurements were performed by two independent observers, and the values were averaged. The intraclass correlation coefficient (ICC) was calculated to evaluate the reliability of measurement results. (ICC for R1 0.935, R2 0.866, d 0.907)

The average values of R1, R2, R1/R2 and d were calculated. A scatter plot including population data and Oxford UKA component data was constructed. SPSS v19.0 software was used for statistical analysis (SPSS, Chicago, Illinois, USA). An independent t test was used to compare the differences between men and women. Dimensions are expressed as mean ± SD values. The Pearson correlation coefficient was calculated to analyze the correlation between R1 and R2. The significance level was set at p < 0.05 in all tests.

## Results

**Fig. 2:**
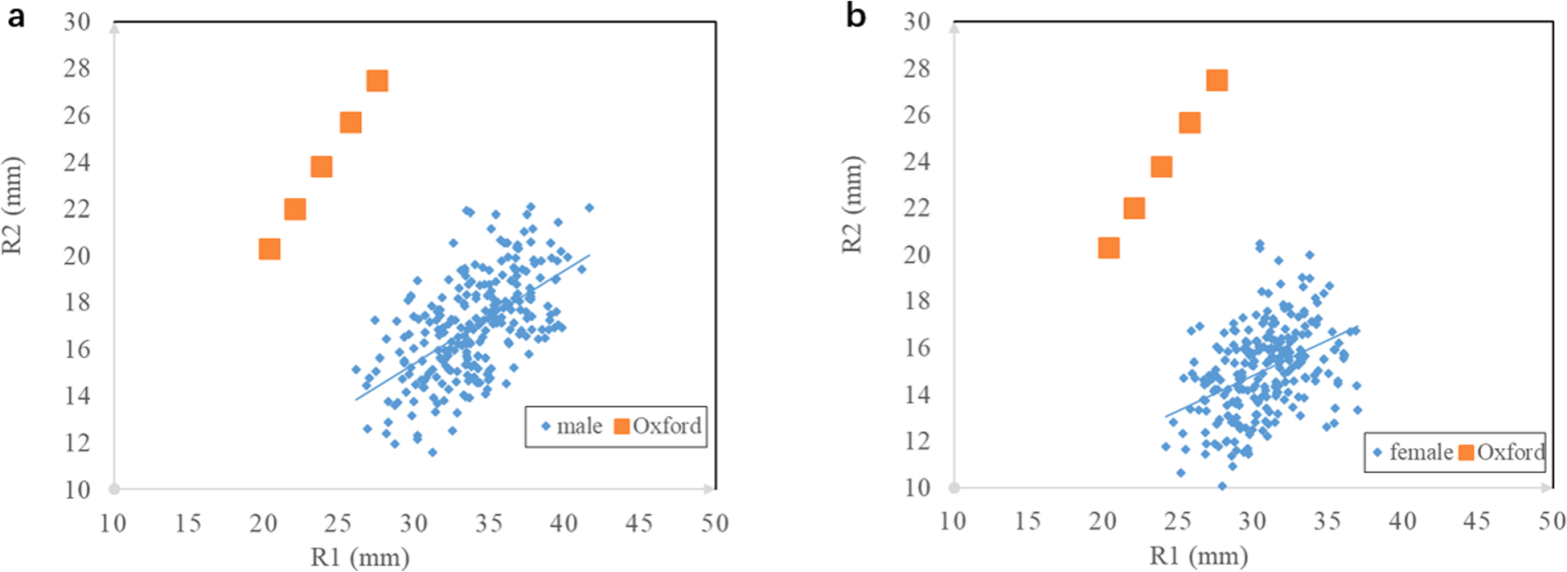
Measurements of the R1 and R2 in the knees of Chinese individuals were compared with similar values in the Oxford component. (a) The measurements of 250 male knees were compared with those of the component. (b) The measurements of 250 female knees were compared with those of the component. The line indicates the average values for the population data

The average values of R1, R2 and d were calculated and compared with previously reported data (Tables 1 and 2). The scatter plot shows the trend of changes in the radii of the distal and posterior articular surfaces of the femoral condyle. The R1/R2 ratio was significantly different from that of the traditional Oxford UKA component (Fig. 2). Moreover, R1, R2 and d differed significantly between men and women (*p* < 0.05). Correlation analysis showed that R1 was positively correlated with R2 (*r* = 0.61, *p* < 0.05).

**Table 1:** Average values of the proximal medial femoral condyle morphological measurements

parameter	male	female	combined	p value
R1	33.9 ± 3.1 mm	30.6 ± 2.6 mm	32.3 ± 3.3 mm	< 0.05
R2	17.0 ± 2.2 mm	15.0 ± 1.9 mm	16.0 ± 2.3 mm	< 0.05
R1/R2	2.01 ± 0.21	2.06 ± 0.26	2.04 ± 0.24	< 0.05
d	15.8 ± 2.7 mm	14.4 ± 2.5 mm	15.1 ± 2.7 mm	< 0.05

**Table 2:** Summary of medial femoral condyle morphological measurements reported by different authors

Author	Population	R1	R2
Zoghi[21]	American	35–42 mm	20 mm
Siu[13]	Canadian	42 mm	22.4 mm
Iwaki[15]	British	32 mm	22 mm
Nuno[14]	Canadian	35 mm	18.9 mm
Chen[5]	Chinese	31.4 ± 2.9 mm	18 ± 1.6 mm
Our study	Chinese	32.3 ± 3.3 mm	16.0 ± 2.3 mm

## Discussion

The most important finding of this study was that the anatomical parameter data (shape) of the medial femoral condyle in a Chinese population were established. This study not only aimed to obtain the parameters of a single femoral condyle but also to use simple geometric parameters to describe the shape of the femoral condyle in the population as a whole based on measurement of multiple samples. In component design, representative geometry of the knee joint is usually needed, and the irregular joint geometry and differences between the test pieces must be considered. Because a shape-matched component is necessary for successful knee replacement surgery, our findings provide the necessary basis for the future design of UKA components. Although studies have investigated the anthropometry of the medial femoral condyle in the sagittal plane [5, 14, 15, 21], this study is the first to use large-sample MRI data for analysis. Our results are expected to facilitate improvements in the clinical efficacy of UKA.

Currently, the design of the Oxford femoral component constitutes a sphere with a single radius [16]. This design feature is significantly different from the normal knee joint in terms of anatomical morphology and geometric structure, and these differences may adversely affect knee joint kinematics, resulting in changes in knee joint function [15]. However, from an engineering perspective, these design compromises may be necessary to maintain the ability of the polyethylene bearing to slide along the surface of the tibial component, increase the stress area and maintain the stability of the component. The material properties of this component are different from those of natural articular cartilage and the meniscus. The arrangement and orientation of the distal femur geometry are assumed to be naturally evolving to provide optimal motor function of the knee joint in terms of muscle strength, ligament restraint, and the material properties of bone, meniscus, and articular cartilage [19]. The purpose of modern UKA is to improve defective knee joint function. Not only must normal limb alignment be restored, the internal geometry should also be aligned or placed optimally with respect to the functional axis of the knee joint so that it can work in harmony with the remaining soft tissues [22]. More importantly, due to the limitations of surgical techniques and equipment, orthopedic surgeons make the final decision on the appropriate placement of the femoral component. The femoral component should be as close to the normal knee joint surface as possible to restore limb alignment and functional joint movement. Research on the anatomical parameters of the femoral shape will facilitate the design of a UKA component that is more suitable than the current component for maintaining kinematic function in the Chinese population, and more ideal clinical results are anticipated.

Comparison of the scatter plots showed that the radius of the distal articular surface of the medial femoral condyle is far larger than that of the posterior articular surface. In this study, the R1 and R2 values were 32.3 ± 3.3 mm and 16.0 ± 2.3 mm (combined), respectively, smaller than the corresponding values in the Western population [15, 19–21], but similar to those in a Chinese population[4]. This finding suggests that there are differences in femoral anatomical parameters between Chinese and Western populations, which are reflected in the observation that the UKA femoral components designed based on the Western population cannot perfectly fit Chinese individuals.

The results of this study showed significant differences in the measurement data between men and women. The radii of both the distal and posterior femoral condyles are larger in men; this difference may be related to variations in bone length between men and women, because the average height of men is greater than that of women [23]. This observation suggested that larger femoral components may be more suitable for male surgical patients, consistent with our previous experience. We found that the average distance between the center of the distal surface and the center of the posterior surface was 15.8 ± 2.7 mm in men and 14.4 ± 2.5 mm in women, indicating that the center of rotation moves forward during flexion. A UKA component designed with a single center of rotation will not be able to completely restore the anthropometry of the knee joint required for a normal gait, and this limitation should be considered in future component design.

This study has some limitations. First, healthy adults were selected as the research objects. UKA is generally performed in the elderly population, but the target population selected in this study was younger, possibly leading to bias. However, due to osteoarthritis, osteophyte formation, wear and other pathological changes, the morphology of the femoral condyle is altered in elderly individuals and is unsuitable for the measurement of anatomical data. Second, only the radius of the femur was measured only the sagittal plane in this study, and measurements in the coronal plane and cross section of the femur are lacking. Third, the study population was limited to the Chinese population. The data in this study may be representative of typical Chinese knee joints, and further studies may be needed to determine the anatomical differences between different populations.

## Conclusions

In this study, the radii of the distal and posterior articular surfaces of the medial femoral condyle and the distance between the two centers of rotation were measured in order to provide guidance for the design of UKA more suitable for Chinese population. In the UKA design, the relationships between the radii of the distal and posterior articular surfaces of the medial femoral condyle should be taken into account.

## Data Availability

The datasets used and/or analyzed during the current study are available from the corresponding author on reasonable request.

## Abbreviations

UKA: Unicompartmental knee arthroplasty
OA: Osteoarthritis
TKA: Total knee arthroplasty
MRI: Magnetic resonance imaging
